# Where and how many: evolutionary diversification of a molecular switch regulating flagellar patterns

**DOI:** 10.1128/jb.00329-25

**Published:** 2025-12-08

**Authors:** Gert Bange, Georg Hochberg, Kai Thormann, Anita Dornes

**Affiliations:** 1 Center of Synthetic Microbiology, Philipps-University Marburg 539292 , Marburg, Germany; 2 Department of Chemistry, Philipps-University Marburg 9377, Marburg, Germany; 3 Max Planck Institute for Terrestrial Microbiology 28310, Marburg, Germany; 4 Department of Biology, Philipps-University Marburg 9377, Marburg, Germany; 5 Department of Microbiology and Molecular Biology, Justus-Liebig University Giessen 9175, Giessen, Germany; Geisel School of Medicine at Dartmouth, Hanover, New Hampshire, USA

**Keywords:** flagellum, nucleotide-binding proteins, macromolecular machine, evolution, regulatory network, protein-protein interaction (PPI), type 3 secretion system, signal recognition particle (SRP), Min system, motility

## Abstract

Flagella are rotating organelles of locomotion that enable bacteria to navigate their environments. They are positioned at various locations and in differing numbers across the bacterial surface, a characteristic known as the “flagellation pattern.” Surprisingly, many of these diverse patterns are regulated by a conserved molecular switch composed of the GTP-binding protein FlhF and the ATPase FlhG, with FlhG stimulating the GTPase activity of FlhF. The evolutionary origins of FlhF and FlhG can be traced to the signal recognition particle (SRP) system and the MinD-dependent cell division machinery, respectively. Here, we review current knowledge on the mechanisms by which the conserved FlhF/FlhG switch controls flagellation patterns across different bacterial species. This system exemplifies how evolution repurposes ancient cellular machineries to control new functions, highlighting the adaptability of protein-based regulatory networks.

## THE BACTERIAL FLAGELLUM—AN ANCIENT AND CONSERVED MOTOR STRUCTURE

**Fig 1:**
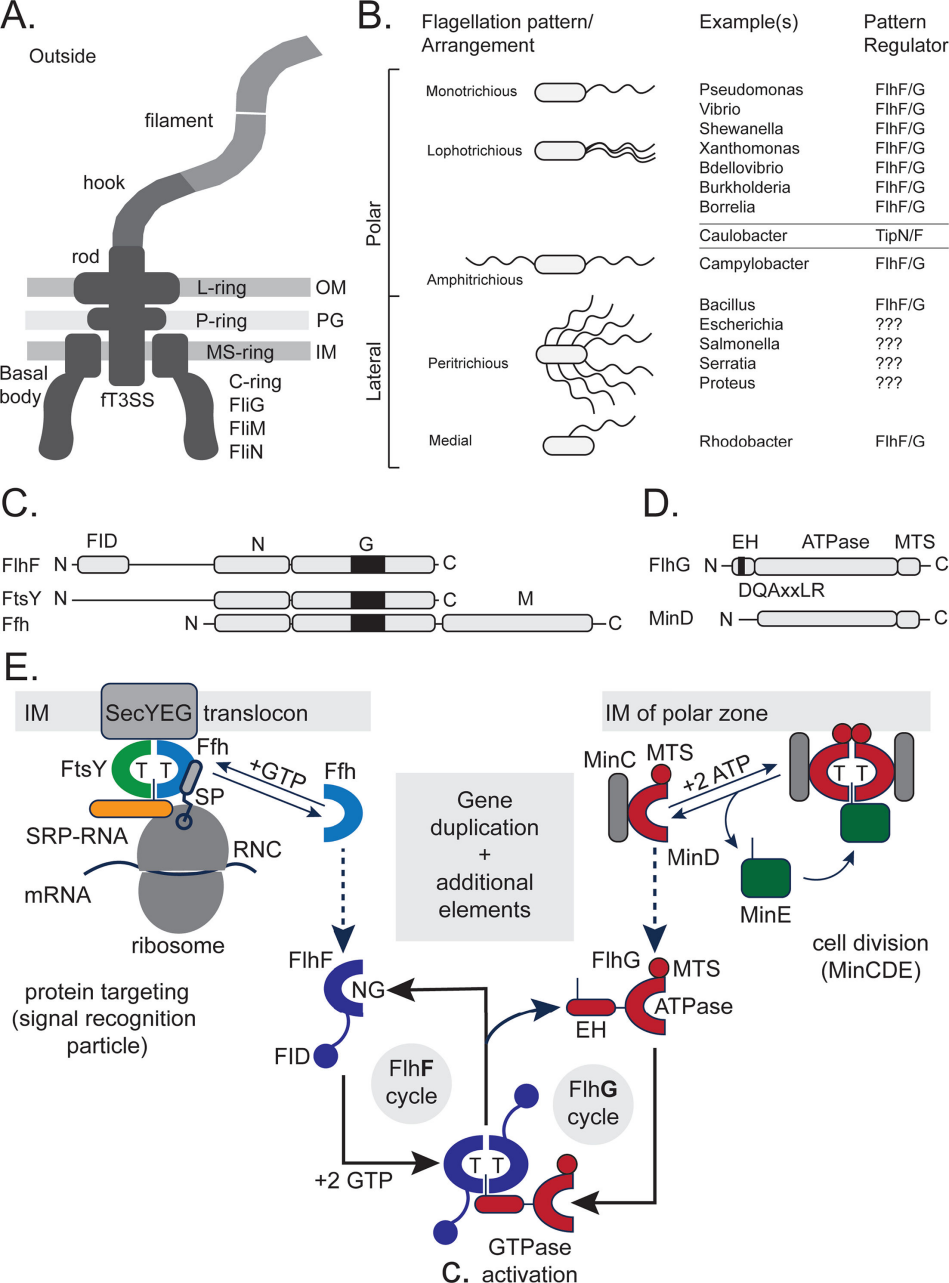
FlhF-FlhG regulate diverse flagellation patterns. (**A**) Scheme of a bacterial flagellum of a Gram-negative bacterium. The abbreviations are: “IM”: inner membrane, “PG”: peptidoglycan, “OM”: outer membrane; “MS”: membrane-supramembrane; and fT3SS: flagellar type 3 secretion system. (**B**) Overview of the different flagellation patterns, examples of bacterial species, and the respective flagellation pattern regulator. A question mark indicates that the system is unknown. (**C**) Domain architectures of the SRP-GTPases FlhF, FtsY, and Ffh. (**D**) Domain architectures of FlhF and MinD. For (**C and D**), the figures are drawn to amino acid scale and individual domains are indicated. The “Ns” and “Cs” indicate the N- and C-termini, respectively. (**E**) The FlhF-FlhG system evolved from the SRP protein-targeting pathway and the MinD-based cell division machinery. *Left side*: the SRP system, composed of the GTPases Ffh (light blue) and FtsY (green), coordinates ribosome-nascent chain complexes (RNCs, gray) bearing signal peptides (SPs) with available SecYEG translocons (gray) in the inner membrane (IM). *Right side*: MinD (red) forms ATP-dependent homodimers that associate with the inner membrane via its membrane-targeting sequence (MTS). Together with MinC (gray) and MinE (green), it ensures the correct positioning of the FtsZ ring at midcell. *Middle*: FlhF (dark blue) and FlhG (red) occurred as gene duplications and present a new regulatory circuit enabling the spatial-numerical regulation of flagellation patterns via a new network of interaction partners.

The bacterial flagellum is a complex, rotary motor that powers bacterial motility. It consists of three main parts: the basal body, the hook, and the filament (Fig. 1A) (reviewed in: [1–3]). The basal body anchors the flagellum to the cell envelope and contains the motor that drives rotation. The hook connects the basal body to the filament and acts as a flexible joint. The filament, composed primarily of flagellin proteins, functions as a long, helical propeller that rotates to drive bacterial motility. In Gram-negative bacteria, the basal body spans both the inner and outer membranes and includes multiple ring structures (the MS-, P-, and L-rings; Fig. 1A). The membrane-supramembrane (MS) ring and cytoplasmic (C) ring are essential structural elements at the base of the bacterial flagellum, positioned within the C membrane and cytoplasm, respectively (Fig. 1A). The MS-ring, primarily composed of the protein FliF, serves as a scaffold for the assembly of the flagellar motor and forms a central pore that accommodates the flagellar type III secretion system (fT3SS) core complex, which mediates the export of flagellar components during assembly. The C-ring, made of the proteins FliG, FliM, and FliN, is essential for torque generation and rotational switching. FliF directly interacts with the C-ring protein FliG, establishing the interface between the MS- and C-rings. In *Bacillus subtilis*, FliY replaces FliN but is not a direct ortholog, as it contains additional EIDAL and CheC-like domains (4). Moreover, *Helicobacter pylori* and *Campylobacter jejuni* encode both FliN and FliY, which are functionally distinct proteins. Taken together, these rings anchor the flagellum to the cell membrane and constitute the rotor, which interacts with the transmembrane stator complexes (MotAB/PomAB). These stators transduce ion motive force into torque on the C-ring to drive rotation (reviewed in references 5, 6). Their coordinated function is crucial for bacterial motility and chemotaxis (7). Taken together, the flagellum plays a vital role in bacterial motility, and its highly conserved structure across diverse species highlights its evolutionary significance as a fundamental motility apparatus. In contrast, Gram-positive bacteria have a slightly “simpler” architecture, with the basal body embedded only in the thick peptidoglycan layer and the membrane, usually involving fewer ring structures (not shown; compare to reference 8). Despite these structural differences, the basic mechanism of flagellar rotation and chemotactic navigation appears highly conserved.

## LOCALIZATION AND NUMBER: SPATIAL ORGANIZATION OF FLAGELLA ON THE CELL SURFACE

Flagella, visible as long helical appendages on the bacterial cell envelope, were recognized early in physiological studies as distinguishing features of bacterial species, as exemplified in Leifson’s Atlas of Bacterial Flagellation (9). Their number and spatial arrangement on the bacterial surface - known as flagellation patterns - vary widely among species and are important taxonomic features, visualized historically by electron microscopy and staining techniques. Common flagellation types include monotrichous (a single polar flagellum), lophotrichous (a tuft of polar flagella), amphitrichous (a flagellum at each pole), and peritrichous (flagella distributed over the entire cell surface, typically excluding the cell poles) (Fig. 1B). These flagellation patterns remain stable within bacterial taxa and are often used for classification, for example, *B. subtilis* and *Escherichia coli* exhibit peritrichous flagella, *Vibrio cholerae* is monotrichous, and *C. jejuni* displays an amphitrichous flagellation pattern (Fig. 1B). Beyond their value in classification, flagellar organization directly influences bacterial motility strategies, including swimming, swarming, and tumbling (10–12), which in turn affect colonization, nutrient acquisition, and host interactions. To maintain these motility patterns, flagellation must be precisely regulated and reproduced during each round of cell division, ensuring that daughter cells contain the correct flagellar positioning and number. Despite the fundamental role of these structures, the molecular mechanisms governing their spatial control and duplication remain incompletely understood. Emerging evidence suggests that flagellar placement is coordinated by cellular polarity cues and landmark proteins that define specific assembly sites, ensuring reproducible flagellar localization and consistent motility behavior across generations.

## A CONSERVED MOLECULAR SWITCH ORCHESTRATES A RANGE OF DIFFERENT FLAGELLATION PATTERNS

Over the past two decades, compelling evidence has shown that two conserved nucleotide-binding proteins, FlhF and FlhG, play a central role in regulating flagellar placement and number in various bacterial species (reviewed in reference 13). Although FlhF and FlhG are highly conserved among flagellated bacteria - and even co-transcribed - they regulate a wide range of flagellation patterns, including monotrichous, amphitrichous, medial, and peritrichous types (Fig. 1B).

*The GTPase FlhF* is essential for the correct placement, initiation, and promotion of flagellar assembly in many differently flagellated bacteria such as *V. cholerae* (14), *Borrelia burgdorferii* (15), *V. alginolyticus* (16), *Pseudomonas putida* (17, 18), *P. aeruginosa* (19), *Xanthomonas oryzae* (20), *H. pylori* (21, 22), *Leptospira* (23), *C. jejuni* (24, 25), *Shewanella putrefaciens* (26), *Shewanella oneidensis* (27, 28), *Burkholderia cenocepacia* (29), *Bacillus cereus* (30), and *B. subtilis* (31, 32). FlhF is a multidomain protein comprising an N-terminal B-domain that contains the FliG-interacting domain (FID) and a long, likely unstructured linker connecting it to the NG domain. The latter consists of an N-terminal α-helical subdomain (N-domain) and a C-terminal nucleotide-binding GTPase subdomain (G-domain) characteristic of SRP-type GTPases (Fig. 1C). Structural studies have shown that the NG-domain of FlhF forms a GTP-dependent homodimer, and this dimerization is crucial for its function in flagellar biosynthesis and localization (33–36). FlhF’s GTP-binding and hydrolysis activities are required for proper flagellar number and positioning in many species, although the specific requirement for GTPase activity varies across them. Mutations that disrupt GTP binding or impair the GTPase cycle led to abnormal flagellation, such as misplaced or absent flagella (25, 37–44).

*The ATPase FlhG* is also known as FleN (45), YlxH (46), MinD2 (47), or MotR (48). The protein is relevant for the numerical regulation of polar flagella (reviewed in references 13, 18, 39, 49), in species such as *V. cholerae* (14), *V. alginolyticus* (16, 50), *P. aeruginosa* (45), *C. jejuni* (51), *H. pylori* (22), and *S. putrefaciens* (52). In *B. subtilis*, FlhG serves in the correct spatial distribution of the peritrichous flagella (53, 54). FlhG can form functionally relevant homodimers that specifically rely on ATP (52, 55). In contrast to its monomeric form, FlhG homodimers can interact with the anionic phospholipids of the cytoplasmic membrane via a membrane-targeting sequence (MTS) located at their C-termini (52, 56, 57) (Fig. 1D). These features enable nucleotide-dependent cycling between its monomeric and dimeric states, allowing FlhG to shuttle between the cytoplasm and the C membrane (52). FlhG features an N-terminal helical extension - termed the enhancer helix (EH) - which contains the conserved “DQAxxLR” motif (where “x” represents any amino acid) (Fig. 1D) (26, 42, 51, 52, 58). The EH stimulates the GTPase activity of FlhF through its conserved glutamine residue, which functions as a co-catalytic element (42). This interaction promotes the transition of FlhF from an active dimer to an inactive monomer. The FlhF-FlhG regulatory circuit, conserved across diverse bacteria, ensures precise control of flagellar number and positioning, enabling species-specific motility patterns and contributing to pathogenicity (23, 59, 60).

## EVOLUTIONARY ORIGIN OF THE FLHF-FLHG REGULATORY CIRCUIT

Accurate targeting of macromolecular machines is essential for proper cellular function. Equally important is maintaining the correct number of these complexes, prompting the question of how cells evolved mechanisms to coordinate both spatial and numerical control. The FlhF-FlhG regulatory circuit offers a compelling example, providing insights into the evolutionary origins of its components. Governing both the localization and number of flagella across diverse flagellation patterns (Fig. 1B), this circuit serves as an ideal model for studying how regulatory networks evolve to yield distinct functional outcomes.

FlhF belongs to the signal recognition particle (SRP) GTPase family (31, 33, 61), a protein family with only two more members, namely Ffh and FtsY. Although further research is certainly needed, it has been proposed that FlhF originated from a gene duplication event involving the SRP-GTPase Ffh (42) (Fig. 1C and E). Ffh associates with a small non-coding RNA to form the SRP, which recognizes signal sequences on nascent polypeptides. The SRP receptor FtsY mediates their delivery to the membrane for insertion or secretion (reviewed in references 61, 62). In short, Ffh and FtsY form a GTP-dependent, pseudosymmetric heterodimer through their NG-domains, which guide ribosome–nascent chain complexes (RNCs) with signal peptides to an available SecYEG translocon for proper membrane insertion or protein secretion (Fig. 1E). GTPase activities of the Ffh-FtsY complex are coordinated by the SRP-RNA, which accelerates their complex formation and promotes reciprocal GTP hydrolysis during protein targeting via a conserved cytidine base (33, 63–66). Similarly, FlhF forms a structurally analogous, GTP-dependent homodimer. Both SRP-GTPase homo- and heterodimers assemble a composite active site that is biologically unique due to the specific “head-to-tail” arrangement of the two GTP molecules within it (33). However, unlike the SRP-RNA-mediated acceleration of GTPase activity in the Ffh-FtsY heterodimer, a conserved glutamine within the “DQAxxLR” motif of the EH in FlhG fulfills a similar stimulatory role in the FlhF homodimer by functionally replacing the catalytic cytidine of SRP-RNA (42) (Fig. 1E). This demonstrates that FlhG functions as a protein-based analog of SRP-RNA, stimulating FlhF GTPase activity through direct interaction (42). This substitution highlights the mechanistic flexibility of SRP-related GTPases and illustrates how distinct molecular components can evolve to perform analogous regulatory roles.

A similar observation holds for FlhG, which is a single domain protein of the SIMIBI clade of P-loop containing, nucleotide-binding proteins (“SIMIBI” after SRP, MinD, and BioD) (47, 67) (Fig. 1D). FlhG proteins share substantial structural and sequence homology with the MinD ATPase (52, 55), which is central for the determination of the cell division site in rod-shaped bacteria (reviewed in reference 68). Similar to MinD, FlhG can alternate between monomeric and dimeric states, with dimerization being ATP-dependent (52, 55). In its dimeric form, FlhG interacts with anionic membrane phospholipids through its C-terminal MTS (52, 56, 57) (Fig. 1D and E). In addition to its similarity with MinD, FlhG has evolved a unique N-terminal α-helix containing the conserved “DQAxxLR” motif, which is essential for stimulating the FlhF homodimer (Fig. 1E). Taken together, the structural and functional parallels between SRP-GTPases, the MinD system, and the FlhF-FlhG pair highlight how ancient molecular frameworks have been repurposed—laying the groundwork to trace the evolutionary emergence of this regulatory circuit across bacteria.

## FLHF AND FLHG COORDINATE FLAGELLAR GENE EXPRESSION, ASSEMBLY, AND POSITIONING IN MONOTRICHOUS FLAGELLATES

It is striking that, despite extensive research, our understanding of how FlhF ensures correct flagellar placement and efficient initiation of assembly across diverse flagellation patterns remains incomplete. Early studies implicated FlhF in recruiting the basal body protein FliF to the cell pole via interactions with the flagellar C-ring (44, 69–71), yet the precise molecular mechanism remained elusive.

**Fig 2:**
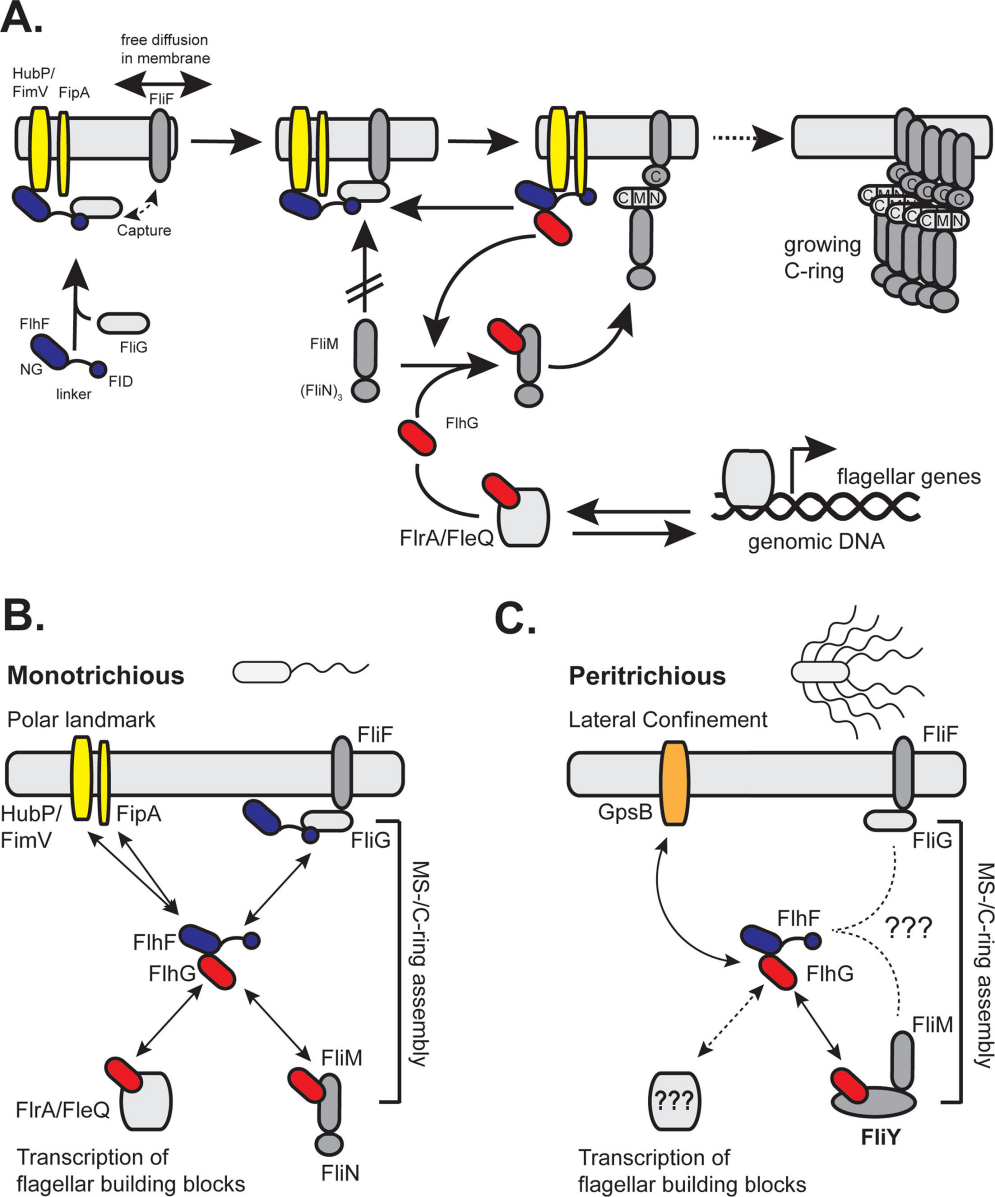
FlhF-FlhG-mediated regulation of different flagellation patterns. (**A**) Model of flagellar assembly and regulation in monotrichous bacteria. FlhF (dark blue) and FlhG (red) control the localization and number of flagella through interactions with structural and regulatory components. Color code: HubP, FimV, FipA (yellow), MS-ring protein FliF (light gray), FliG (white), FliM/FliN (light gray), and transcriptional regulator FlrA/FleQ (gray). Further details are described in the main text. (**B and C**) Overview of known and proposed interaction networks of the FlhF-FlhG system in different flagellation patterns. (**B**) In monotrichous bacteria, FlhF is recruited to the pole via polar landmark proteins (HubP/FimV and FipA) and captures FliF to initiate MS/C-ring assembly. FlhG regulates both structural assembly and gene expression through interactions with FliM and FlrA/FleQ. (**C**) In the peritrichous system of *B. subtilis*, GpsB (orange) may act as a lateral confinement factor for flagellar placement. While FlhF (blue) and FlhG (red) are still involved, their exact spatial cues and regulatory partners remain unclear (indicated by dashed arrows and question marks). In this system, FlhG interacts with FliY (dark gray), a functional homolog of FliN.

A recent study offers a mechanistic framework for how FlhF mediates polar flagellum localization in polarly flagellated bacteria such as *S. putrefaciens* and *Vibrio* species. The multidomain FlhF protein (Fig. 1C) appears to function as a molecular tether that links the polar landmark protein HubP to the developing flagellum (37). Specifically, the NG-domain of FlhF binds the C-terminal domain of HubP, while a newly defined N-terminal region—termed the FliG Interaction Domain (FID)—engages with the C-ring protein FliG. This dual interaction likely enables FlhF to retain nascent FliF proteins at the cell pole (Fig. 2A), potentially capturing them after SRP-dependent co-translational insertion into the membrane (61, 62, 72, 73). Notably, deletion of the FID results in mislocalized flagella, supporting a “diffusion-capture” model for early flagellar assembly (37).

Although HubP is central to this model, its role varies among species. In *V. cholerae*, *V. parahaemolyticus*, and *V. alginolyticus*, deletion of *hubP* leads to distinct and sometimes opposing effects on flagellation (74–76), suggesting evolutionary divergence in polar localization pathways. The recent discovery of the conserved membrane protein FipA, which interacts with FlhF (75), adds another layer of complexity. In species such as *P. putida*, *V. alginolyticus*, and *S. putrefaciens*, FipA and HubP (or its homolog FimV) appear to function redundantly or cooperatively in recruiting FlhF to the pole.

Although its exact role is unclear, FlhF-bound FliG cannot interact with its C-ring partners FliM and FliN (Fig. 2A) (37). This FlhF-mediated block is relieved by FlhG, which stimulates FlhF’s GTPase activity and interacts with FliM/N via the N-terminus of FliM (13, 52), thus permitting C-ring completion (Fig. 2A). Thus, interaction between FlhG and the GTPase FlhF may function as a regulatory checkpoint during flagellar assembly, particularly at the stage of C-ring formation where FliM/N subunits are incorporated into FliG (Fig. 2A) (37).

In monotrichous flagellates, FlhG (also known as FleN) has also been shown to directly interact and modulate the ATPase activity of FlrA (aka FleQ), a master transcriptional regulator of flagellar gene expression. This interaction inhibits the ATPase activity of FlrA, thereby repressing the transcription of flagellar and motility genes (45, 58, 77, 78). Thus, FlhG plays a dual role in both structural assembly, by interacting with FlhF and contributing to C-ring formation, and transcriptional regulation via the master regulator FlrA (or FleQ in *Pseudomonas*). Interestingly, *flhG* mutants in monotrichous species are hyperflagellated rather than aflagellate, likely due to loss of FlhG-mediated repression of FlrA, which leads to elevated flagellar gene expression that compensates for assembly defects (16, 45, 58). This suggests that FlhG can relay the assembly status of the flagellar C-ring to influence the transcription of flagellar genes, including those controlling flagellar number and positioning. As the C-ring assembles, FlhG localizes to the cell pole, modulating its interactions with both FlrA and FlhF. These findings refine the traditional model of flagellar gene regulation as a strictly hierarchical transcriptional cascade, pointing instead to a more integrated system where structural assembly and gene expression are tightly coupled. Finally, insights from the roles of FlhF and FlhG in flagellar assembly in monotrichous bacteria will help guide future studies aimed at uncovering alternative mechanisms or novel molecular factors that confer spatial specificity to FlhF in systems lacking known polar landmarks (see next chapter).

## DIVERSIFICATION OF FLHF-FLHG INTERACTIONS IN NON-MONOTRICHOUS FLAGELLATION

As outlined above, FlhF and FlhG can contribute to all known flagellation patterns (Fig. 1B). While the regulatory mechanisms in monotrichous bacteria are beginning to become a complete picture, much less is known about how these proteins function in other flagellation types. It seems that in non-monotrichous bacteria, diversification of FlhF-FlhG interactions, including changes in binding partners and regulatory networks, enables adaptation to different spatial and numerical patterns of flagella. Comparative studies across diverse bacterial species begin to uncover the molecular basis of these adaptations. In the following paragraphs, we will summarize what is already known:

### Landmark proteins and confinement factors

Landmark proteins and confinement factors are key spatial regulators that define specific sites within the bacterial cell, such as the poles or midcell. Landmark proteins recruit functional partners to these locations, while confinement factors restrict protein movement, ensuring precise localization of cellular machinery like the flagellar system. In monotrichous flagellates, the landmark proteins HubP/FimV and FipA play a central role in establishing the polar site for flagellar assembly by interacting with FlhF, thereby restricting MS- and C-ring formation to the cell pole (see previous chapter; Fig. 2B) (37). How landmarking and confinement operate in flagellation patterns beyond the monotrichous type remains unclear. Recent work has provided insight into the FlhF/FlhG-dependent arrangement of peritrichous flagella in *B. subtilis*: ATP-bound FlhG homodimers interact with the C-terminal domain of GpsB, a cell cycle regulator that recruits the peptidoglycan synthase PBP1 (*ponA*) to sites of cell wall elongation (54) (Fig. 2C). Moreover, FlhG was previously shown to interact with the C-ring protein and FliN homolog FliY (52) (Fig. 2C). The dual interaction of FlhG with both GpsB and FliY, along with GpsB’s ability to bind PBP1, supports a model in which FlhG coordinates flagellar assembly with regions of active cell wall synthesis. These findings raise the possibility that coupling flagellar assembly to cell wall growth via landmarking and confinement may be a broader strategy in bacteria, warranting further investigation across diverse flagellation systems.

### The flagellar C-ring proteins

Early papers have already suggested involvement of the flagellar basal body proteins in the molecular functioning of FlhF and FlhG. In a hallmark study, Green et al. showed that recruitment of the MS-ring protein FliF to the pole required FlhF (44). The notion was further specified by showing that FlhF does so by a diffusion-capture mechanism involving the C-ring FliG and the landmark proteins HubP and FipA (37, 75) (Fig. 2B). The specific roles of FipA and HubP remain to be fully elucidated. Furthermore, it is still unclear whether the establishment of alternative flagellation patterns, beyond the monotrichous type, depends on interactions between FlhF and the C-ring protein FliG, or whether analogous diffusion-capture mechanisms involving FliF are involved.

Similarly, FlhG has been shown to interact with the C-ring components three proteins FliG, FliM, and FliN (or its homolog FliY) (Fig. 2B and C). In monotrichous flagellates, FlhG interacts specifically with FliM, but not with FliN (52) (Fig. 2B). In contrast, in the peritrichously flagellated *B. subtilis*, FlhG interacts with the FliN homolog FliY, but not with FliM (reviewed in reference 13) (Fig. 2C). It remains unclear whether FlhG interacts with specific proteins in amphitrichous, lophotrichous, and lateral flagellation systems, and if so, which ones. Determining whether these interactions are conserved or lineage-specific is crucial to understanding how FlhG regulates flagellar number, positioning, and gene expression across diverse bacteria.

### Transcription factors

A body of literature describes the interaction of FlhG with the FlrA/FleQ-type transcription factors. FlhG and its homolog FleN control flagellation by modulating the activity of the regulatory proteins FlrA and FleQ, respectively, through direct interactions (58, 79, 80). However, these interactions have so far been characterized and seem to exist only in monotrichous flagellates (Fig. 2B). Virtually nothing is known about whether FlhG or FlhF interact with, or modulate, the transcriptional machinery that governs flagellar gene expression in other flagellation systems (e.g., Fig. 2C). It remains an open question whether these proteins engage in regulatory crosstalk with master transcriptional regulators beyond FlrA or FleQ, or whether alternative regulatory pathways have evolved. Thus, future studies should aim to identify potential transcriptional interaction partners of FlhG and FlhF across diverse bacterial species and flagellation modes.

## THE CURIOUS CASE OF THE γ-PROTEOBACTERIA

Phylogenetic analysis reveals that *flhF* and *flhG* co-occur in about 30% of bacterial genomes across diverse lineages. Remarkably, they are consistently encoded together, with *flhG* directly downstream of *flhF*, despite extensive flagellar operon rearrangements (42). Their absence in some motile species may reflect either the emergence of newer regulatory systems (e.g., in the α-proteobacterium *Caulobacter crescentus*) (13, 81, 82) or genome streamlining that eliminates (polar) flagellation.

Many enterobacteria, such as *E. coli* and *Salmonella*, lack the genes encoding FlhF and FlhG, yet display a peritrichous flagellation pattern (Fig. 1C). This peritrichous organization appears to be the result of a horizontal gene transfer event, in which the entire flagellar and chemotaxis operon from betaproteobacteria was transferred into enterobacteria, replacing the ancestral gammaproteobacterial flagellar system (73, 74). The *flhF* and *flhG* genes were either not included in the transferred operon or were lost shortly thereafter, leading to the apparently uncoordinated flagellar placement observed in enteric bacteria like *E. coli* and *S. enterica*. In contrast, other non-enteric γ-proteobacteria, such as *Shewanella*, have retained their ancestral flagellar gene cluster—including *flhf* and *flhG*—and produce polar flagella (Fig. 1C). These findings raise the question of whether peritrichous flagellation in enteric γ-proteobacteria lacking *flhF* and *flhG* is regulated or random.

A recent study in *E. coli* showed that the MinDE system, known for positioning of the bacterial cell division ring (Z-ring), also represses *fliA* transcription via interaction with the transcription factor AtoS, limiting flagellar gene expression (83). Loss of MinD lifts this repression, increasing FliA levels and flagella production. These findings clearly show that the FlhG homolog MinD can take over regulatory functions in flagella biosynthesis. *Vice versa*, *C. jejuni*, which lacks MinD, relies on FlhG for numerical control of flagellation and for ensuring accurate, symmetrical division (51, 84) (Fig. 1B). However, the precise underlying mechanisms by which both proteins coordinate motility and cytokinesis in each of the species remain to be fully elucidated. From an evolutionary perspective, this functional interchangeability suggests a shared ancestral role for MinD and FlhG, followed by lineage-specific specialization. The divergence of these systems across bacterial phyla may reflect adaptations to distinct cellular architectures, lifestyles, or ecological niches.

Whether *E. coli* has a FlhF equivalent remains unclear. No homolog has been identified, but other GTPases or membrane proteins may play a similar role. Notably, the SRP receptor FtsY in *E. coli* has a unique, negatively charged N-terminal A-domain—a feature uncommon outside γ-proteobacteria (G.B., personal observation; Fig. 1C). This could represent a lineage-specific adaptation. Alternatively, *E. coli* may use entirely different cues—such as cell geometry or unknown protein complexes—for flagellar placement. Further studies are needed to uncover the molecular basis of its flagellar organization.

## CONCLUDING REMARKS

The FlhF-FlhG system is a powerful example of how bacteria reuse ancient proteins for new roles. Once part of protein-targeting and cell division systems, FlhF and FlhG now control where and how many flagella are built, helping bacteria adapt their motility to different environments. Though the core mechanism is conserved, the system has evolved to work with diverse cellular components in different species. To fully understand this flexibility, future research should explore how flagellar regulation connects to other cellular processes like division and polarity. Tools like evolutionary genomics, advanced imaging, and synthetic biology will be key. Studying this system across more bacterial species may reveal new principles of cellular organization and how complex systems evolve over time.
